# A nationwide cross-sectional survey on factors affecting turnover intention among hospital pharmacists

**DOI:** 10.1186/s12960-026-01052-4

**Published:** 2026-02-07

**Authors:** Junsung Nam, JaeEun Han, Young-Mi Ah, Yun Mi Yu

**Affiliations:** 1 Department of Pharmaceutical Medicine and Regulatory Sciences, Colleges of Medicine and Pharmacy, Yonsei University, Incheon, Republic of Korea; 2 Department of Pharmacy, Ajou University Hospital, Suwon, Republic of Korea; 3 College of Pharmacy, Yeungnam University, 280 Daehak-Ro, Gyeongsan, Gyeongsangbuk-do 38541 Republic of Korea; 4 Department of Pharmacy and Yonsei Institute of Pharmaceutical Sciences, College of Pharmacy, Yonsei University, 85 Songdogwahak-ro, Yeonsu-gu, Incheon, 21983 Republic of Korea

**Keywords:** Hospital pharmacists, Turnover intention, Job embeddedness, Job stress

## Abstract

**Background:**

Hospital pharmacists play a pivotal role in ensuring the safe and effective use of medications, thereby supporting the quality of care and the resilience of health systems. Identifying the factors influencing turnover intention among hospital pharmacists and implementing strategies to maintain an appropriate talent pool can contribute to strengthening public health and improving patient outcomes. This study aimed to identify the factors influencing turnover intention among hospital pharmacists in South Korea and provide implications for strategies to support pharmacist retention.

**Methods:**

A cross-sectional survey was conducted in July 2024 with 592 full-time pharmacists employed in tertiary and general hospitals using proportional stratified sampling by hospital type and region, representing 16.0% of the pharmacists working in these hospitals. The questionnaire incorporated items from Mitchell’s Job Embeddedness theory, the Korean Occupational Stress Scale, and Singh’s turnover intention scale, and underwent expert content validation and exploratory factor analysis. Multivariable linear and logistic regression analyses were performed to determine the factors associated with turnover intention after adjusting for key demographic and institutional characteristics.

**Results:**

Among the 592 respondents, 255 (43.1%) had high turnover intention, with shorter employment durations associated with higher turnover intention scores. Within job embeddedness, factors significantly reducing turnover intention included fit to organization_task, organization-related sacrifice_direct, link to organization_task, and link to community_transverse. Within job stress, the factors that significantly increased turnover intention were lack of rewards, job demand_density, and organizational system_fairness. The identified associations were consistent across both linear and logistic regression models, supporting the robustness of the findings.

**Conclusions:**

To retain skilled professionals, hospitals should improve their reward structures, foster a culture of fairness, and provide targeted support to junior pharmacists. Improving role fit in task assignments may help reduce turnover risk and enhance workforce stability.

**Supplementary Information:**

The online version contains supplementary material available at 10.1186/s12960-026-01052-4.

## Background

Hospital pharmacists play a key role in ensuring safe, effective, and equitable medication use in healthcare institutions, from dispensing to specialized clinical services [1–3]. Providing patient-centered care improves outcomes and health system quality [4–6]. Retaining skilled and competent pharmacists is fundamental to sustaining high-quality services and ensuring medication safety [4, 7]. Nevertheless, hospital pharmacists are increasingly reporting job dissatisfaction, stress, and intention to leave their positions. In previous studies, the turnover rate of hospital pharmacists ranged from 8.6% to 17% [8]. The American Society of Health System Pharmacists Workforce Survey has reported a turnover rate of approximately 11% among hospital pharmacists in the United States [9]. Such shortages increase workloads and medication errors and limit patient-centered care [10–12].

In South Korea, the turnover rate among hospital pharmacists is expected to reach 20% in 2024, which is considerably higher than the rates reported in other countries, highlighting the need for pharmacist-specific evidence [9, 13]. Nevertheless, to our knowledge, very few studies have directly investigated pharmacists’ turnover intention. The only earlier Korean study we identified, conducted more than a decade ago, addressed job stress and satisfaction, but did not investigate turnover intention [14]. By contrast, the turnover among physicians and nurses has been extensively examined, highlighting a substantial evidence gap for hospital pharmacists in the Korean healthcare system [15–18].

Turnover intention refers to an employee’s voluntary willingness to leave an organization and is a well-recognized precursor of actual turnover [19–21]. Job embeddedness describes the extent to which employees remain attached to their jobs and environment. Mitchell’s Job Embeddedness theory is one of the most influential frameworks in the job embeddedness literature, conceptualizing retention through three dimensions—fit, links, and sacrifice—across two domains: the organization (‘on-the-job’) and the community (‘off-the-job’) [22]. This dual structure proposes that employees remain not only because of workplace compatibility and professional ties, but also because of community relationships and personal or family-related sacrifices associated with leaving [23]. Prior studies have shown that these domains may operate through distinct mechanisms and can shape turnover outcomes independently or jointly, underscoring the importance of examining them separately in turnover research [24]. A meta-analysis of 52 studies demonstrated that both on- and off-the-job embeddedness were strongly and independently associated with turnover intention and actual turnover [25]*.* Mitchell’s Job Embeddedness theory is particularly relevant to hospital pharmacists in South Korea, whose retention is shaped by organizational conditions (e.g., workload, rewards, fairness, and professional relationships) as well as community and family commitments, aligning the dual embeddedness structure with the aims of this study.

In contrast to job embeddedness, job stress arises when job demands (JDs) exceed coping resources and is influenced by workload, lack of autonomy, interpersonal conflict (IC), job insecurity (JI), and organizational systems (OSs). This harms well-being and serves as a key driver that pushes employees toward withdrawal and turnover [8]. A Taiwanese study showed that organizational climate, stress, and burnout predicted pharmacist retention with 56% accuracy [26]. Comparable challenges have also been documented in the United Kingdom’s National Health Service (NHS), where increasing workload and organizational stressors have been linked to higher work stress and turnover intentions among pharmacists [27] and to greater staff turnover and adverse patient outcomes among nurses and doctors [12]. In this regard, we applied Mitchell’s job embeddedness theory to include ‘off-the-job’ factors to comprehensively assess pharmacist turnover intention. Job stress was also examined to provide a more detailed analysis of the job-related factors.

Building on prior evidence indicating a high turnover among hospital pharmacists in South Korea and limited research on its underlying determinants, this study conducted a nationwide survey to address this gap. To examine the factors influencing turnover intention, we applied the job embeddedness and job stress frameworks to assess how their subdimensions relate to turnover intention among full-time hospital pharmacists in tertiary and general hospitals. We further evaluated whether these associations differed across individual and institutional subgroups.

## Methods

### Study design and population

A cross-sectional survey was conducted in July 2024 among full-time pharmacists working in tertiary and general hospitals nationwide in South Korea. Part-time pharmacists or those working in long-term care hospitals were excluded to ensure sample homogeneity and alignment with the study focus. To improve representativeness, a stratified sampling method was employed, using strata proportional to the regional and hospital-type distributions of hospital pharmacists, as reported by the Korean Society of Health-System Pharmacists (KSHP). Stratum-specific target numbers were determined a priori, and recruitment within each stratum continued until a predefined quota was reached. Approximately 3708 eligible pharmacists working in tertiary and general hospitals were invited to participate through the KSHP. Each invitee received an email containing the study description, a consent form, and a survey link; consent was recorded by a checkbox. All items were mandatory in the Google Forms survey. In total, 592 pharmacists completed the survey, corresponding to an overall response rate of 16.0%. Representativeness was evaluated by comparing the distributions of demographic (sex and age) and institutional (regional location and hospital type) characteristics of respondents with the national KSHP workforce statistics [13, 28, 29].

This study was approved by the Institutional Review Board (IRB) of Yonsei University (IRB number: 7001988-202407-HR-2307-03). All methods complied with the Declaration of Helsinki, and reporting followed the Strengthening the Reporting of Observational Studies in Epidemiology (STROBE) guidelines for cross-sectional studies. The STROBE checklist informed the reporting of key methodological domains, including the sampling strategy, efforts to enhance representativeness and reduce potential selection bias, and the handling of missing data (no missing responses were allowed in the survey) [30].

### Questionnaire composition and measure

The questionnaire included 82 items assessing general characteristics and three key domains: job embeddedness, job stress, and turnover intention. General characteristics included demographic and employment.

The job embeddedness domain included six dimensions: fit to organization (FO), link to organization (LO), organization-related sacrifice (OrS), fit to community (FC), link to community (LC), and community-related sacrifice (CrS) [22]. In accordance with Mitchell’s dual-domain framework, the job embeddedness items were organized into organizational and community domains, reflecting the theoretically consistent aspects of fit, links, and sacrifice. In this study, 37 of the original 41 items were selected based on an expert content validity assessment. The expert panel recommended excluding items that provided an overly global evaluations of the job (e.g., statements suggesting that ‘this job has excellent advantages’), overlapped with information already collected as separate variables (e.g., employment duration at the current position), or represented culturally specific family or community circumstances not uniformly applicable to hospital pharmacists (e.g., spouse’s employment outside the home, family being ‘rooted’ in the local community). The items used a 4-point Likert scale, except for items in the LC dimension, which employed binary formats.

The job stress domain was assessed using the Korean Occupational Stress Scale (KOSS), which is based on the Job Strain model and incorporates the Effort–Reward Imbalance model and Job Content Questionnaire, modified for Korean cultural contexts [31]. The KOSS includes 43 items across eight dimensions: physical environment (PE), JD, job control (JC), IC, JI, OS, lack of reward (LR), and occupational climate (OC). In this study, 35 of the 43 original KOSS items were selected based on expert content validity assessment. The expert panel recommended excluding items that addressed situations that were less central to job stress in hospital pharmacy settings (e.g., sudden and unpredictable changes in work schedules and discomfort with work-related social gatherings) or were related to gender-based workplace climate or workplace hygiene (e.g., perceptions of gender disadvantage, workplace cleanliness, and comfort), as these were not considered key sources of job stress for hospital pharmacists. The panel also recommended excluding items capturing the perceived ease of changing jobs or the risk of job loss (e.g., confidence in finding an equivalent position or concern about losing one’s job), which were considered conceptually overlapping with turnover intention and not well aligned with the relatively stable employment structure of hospital pharmacists. All the retained items were assessed using a 4-point Likert scale.

Turnover intention was measured using three items from the Singh Turnover Intention Scale. During expert content validity assessment, panel members confirmed that these items adequately captured both the cognitive and behavioral components of turnover intention in this population. Each item is rated on a 5-point Likert scale, with higher scores indicating stronger turnover intention [32].

### Validation of the adapted instruments

We validated the adapted instruments via face/content validation and exploratory factor analysis (EFA). Eight hospital pharmacists assessed face validity for clarity [33]. Content validity was evaluated by four experts using a 4-point scale; the content validity index (CVI) guided retention (CVI ≥ 0.80), revision (0.70–0.79), or removal (< 0.70) [34]. EFA tested the internal structure of the adapted instruments in our specific population, consistent with previously validated constructs [35–37]. Details of the statistical methods and model fit indices used for the EFA are provided in the “Statistical analysis” section.

### Hypothesis-driven testing of their associations of turnover intention

#### H1:

Higher levels of JE subdimensions (X1, X2, …) are associated with lower turnover intention.

#### H2:

Higher levels of JS subdimensions (Y1, Y2, …) are associated with higher turnover intention.

#### H3:

These associations differ across subgroups (i.e., age, hospital type, and role).

### Sample size

The minimum required sample size was calculated based on a prior study reporting an odds ratio of 2.0 for turnover intention associated with income dissatisfaction [38]. A two-sided α of 0.05 and 90% power were selected, consistent with common practice in health workforce and organizational research and to ensure adequate sensitivity for detecting moderate associations. Using Epi Info™ 7.2.6.0 (Centers for Disease Control and Prevention, Atlanta, GA), the required sample size was 460; assuming a 20% rate of non-response or incomplete data, the final target was 580. Post hoc power analysis was not performed, in line with methodological recommendations that discourage post hoc calculations based on observed data; instead, robustness was examined through complementary sensitivity analyses, including alternative thresholds for defining high turnover intention.

### Statistical analysis

Descriptive statistics summarize the baseline characteristics. EFA was conducted to examine the internal structure of the adapted instruments within our study population, consistent with the construct frameworks reported in previous validations [36, 39]. Data suitability for factor analysis was confirmed with the Kaiser–Meyer–Olkin measure (≥ 0.7) and Bartlett’s test of sphericity (< 0.001) [35]. Principal component extraction with Varimax orthogonal rotation was used to derive an interpretable factor structure [40]. Factors were identified based on a scree plot and eigenvalues > 1, with items retaining factor loading ≥ 0.4 [37]. Internal consistency reliability was assessed using Cronbach’s α, with coefficients ≥ 0.70 considered acceptable [33].

Primary analyses used multivariable linear regression with continuous predictors adjusted for sex, marital status, department, employment duration, hospital type, and hospital location. Linear regression models estimated regression coefficients (*β*) and 95% confidence intervals (CIs). Variance inflation factors (VIFs) were examined to assess multicollinearity (all VIFs were < 2.0).

Predictors were subsequently median dichotomized to facilitate interpretability and stakeholder use [20, 39, 41], and logistic regression models were used to provide adjusted odds ratios (aORs) with 95% CIs. Given the potential for information loss due to dichotomization, linear regression models served as the primary analytical framework. To evaluate the stability of the associations, sensitivity analyses were performed using alternative thresholds to define high turnover intention (e.g., > 8 or > 10 instead of the median cutoff of 9). Subgroups were defined by the six covariates, with interaction p values testing differences. Statistical significance was defined as *P* < 0.05. All analyses were performed using SAS version 9.4 (SAS Institute Inc., Cary, NC, USA).

## Results

### Study population and baseline characteristics

A total of 592 of 3708 eligible full-time pharmacists in tertiary and general hospitals invited nationwide through the KSHP completed the survey, yielding an overall response rate of 16.0% and subgroup response rates ranging from 14.7% to 17.0% across hospital types and regional subgroups. The demographic (sex and age) and institutional (hospital type and regional location) distributions of the respondents were broadly comparable to those of the national hospital pharmacist population, indicating acceptable sample representativeness (Supplementary Table S1). Among the respondents, 87.5% were women, 48.6% were 30–39 years old, and 49.5% were married. Approximately one-third of the hospitals were located in metropolitan areas. The employment duration was as follows: 34.5% had < 3 years, 32.9% had 3–10 years, and 32.6% had ≥ 10 years. The number of years of hospital practice showed a similar distribution (Table 1).

**Table 1:** Baseline characteristics

Characteristics		Total (N = 592)^*^	Turnover risk	
			Low (N = 337)^*^	High (N = 255)^*^
Demographic				
Sex	Female	518 (87.5)	295 (87.5)	223 (87.5)
	Male	74 (12.5)	42 (12.5)	32 (12.5)
Age, years^†^	< 30	102 (17.2)	47 (13.9)	55 (21.6)
	30–40	288 (48.6)	140 (41.5)	148 (58.0)
	40–50	129 (21.8)	88 (26.1)	41 (16.1)
	≥ 50	73 (12.3)	62 (18.4)	11 (4.3)
Marital status^†^	Unmarried	299 (50.5)	152 (45.1)	147 (57.6)
	Married	293 (49.5)	185 (54.9)	108 (42.4)
Education level^†^	Bachelor’s degree	424 (71.6)	217 (64.4)	207 (81.2)
	Master’s degree or higher	168 (28.4)	120 (35.6)	48 (18.8)
Employment				
Years of hospital practice, years^†^	< 1	55 (9.3)	28 (8.3)	27 (10.6)
	1–3	105 (17.7)	43 (12.8)	62 (24.3)
	3–5	75 (12.7)	32 (9.5)	43 (16.9)
	5–10	125 (21.1)	67 (19.9)	58 (22.7)
	10–20	143 (24.2)	95 (28.2)	48 (18.8)
	≥ 20	89 (15.0)	72 (21.4)	17 (6.7)
Employment duration at the current hospital, years^†^	< 1	76 (12.8)	40 (11.9)	36 (14.1)
	1–3	128 (21.6)	56 (16.6)	72 (28.2)
	3–5	76 (12.8)	35 (10.4)	41 (16.1)
	5–10	119 (20.1)	64 (19.0)	55 (21.6)
	10–20	130 (22.0)	90 (26.7)	40 (15.7)
	≥ 20	63 (10.6)	52 (15.4)	11 (4.3)
Current department^†^	General dispensing	273 (46.1)	129 (38.3)	144 (56.5)
	Specialized compounding	71 (12.0)	39 (11.6)	32 (12.5)
	Clinical pharmacy service	73 (12.3)	42 (12.5)	31 (12.2)
	Medication management	49 (8.3)	33 (9.8)	16 (6.3)
	Pharmacy administration and education	126 (21.3)	94 (27.9)	32 (12.5)
Hospital location	Capital region	246 (41.6)	132 (39.2)	114 (44.7)
	Metropolitan cities	146 (24.7)	86 (25.5)	60 (23.5)
	Provinces	200 (33.7)	119 (35.3)	81 (31.8)
Hospital funding type	Private hospital	396 (66.9)	225 (66.8)	171 (67.1)
	National/Public hospital	196 (33.1)	112 (44.2)	84 (32.9)
Hospital type	Tertiary hospital	327 (55.2)	181 (53.7)	146 (57.3)
	General hospital	265 (44.8)	156 (46.3)	109 (42.7)

^*^Number of respondents (%)

^†^Significant difference between low and high turnover risk groups (Chi-square test, *P* < 0.05)

**Fig. 1:**
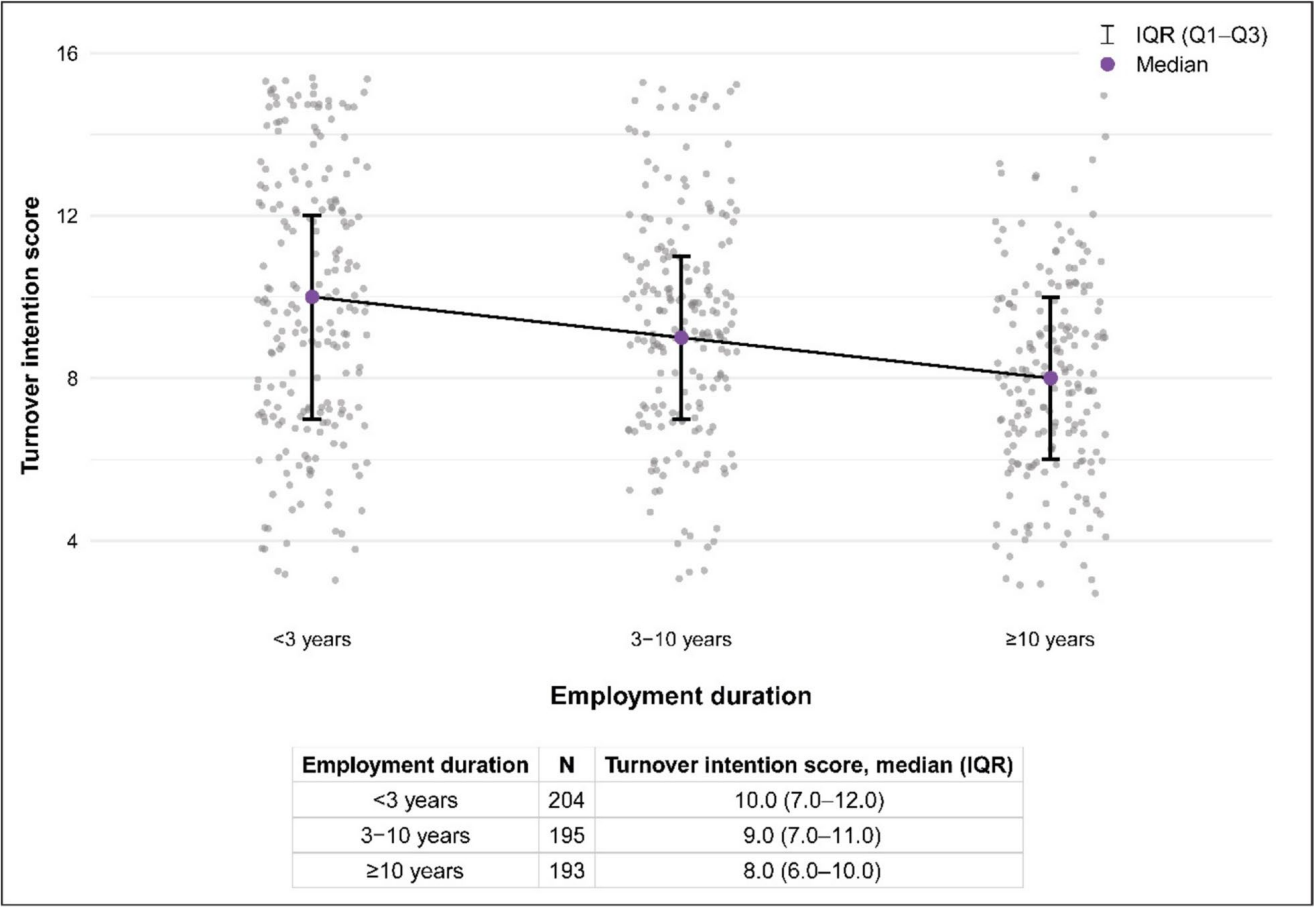
Turnover intention scores by employment duration. Jittered scatterplot of individual turnover intention scores by employment duration category (< 3, 3–10, and ≥ 10 years). Purple dots indicate category-specific medians, and the vertical caps represent the interquartile range. The connected trend line illustrates the overall decreasing pattern of turnover intention with increasing employment duration

Turnover intention scores ranged from 3.0 to 15.0 (median = 9.0), with 255 pharmacists (43.1%) classified as having high turnover intention (score > 9.0). Compared with those reporting low turnover intention (score ≤ 9.0), pharmacists with high turnover intention were more likely to be younger, unmarried, to hold a bachelor’s rather than a graduate degree, and to have shorter employment duration (all *P* < 0.05; Table 1). Turnover intention was the highest among pharmacists with < 3 years of employment and declined with longer employment duration (Fig. 1).

### Construct validity and reliability

Factor analysis revealed that turnover intention items loaded onto a single factor. Within job embeddedness, items for FO, LO, OrS, and LC were split into factors distinguishing task versus peer and longitudinal versus transverse dimensions. Within job stress, JD items are separated into density and pressure factors, and OS items into collaboration and fairness factors. All other job embeddedness and stress dimensions loaded onto a single factor (Supplementary Table S2). Cronbach’s α coefficients indicated acceptable internal consistency for most factors, whereas several subscales—including LC_longitudinal, CrS, OrS_direct, OS_collaboration, and JC—showed α values below 0.70 and should be interpreted with caution (Supplementary Table S3).

### Factors affecting turnover intention

**Fig. 2:**
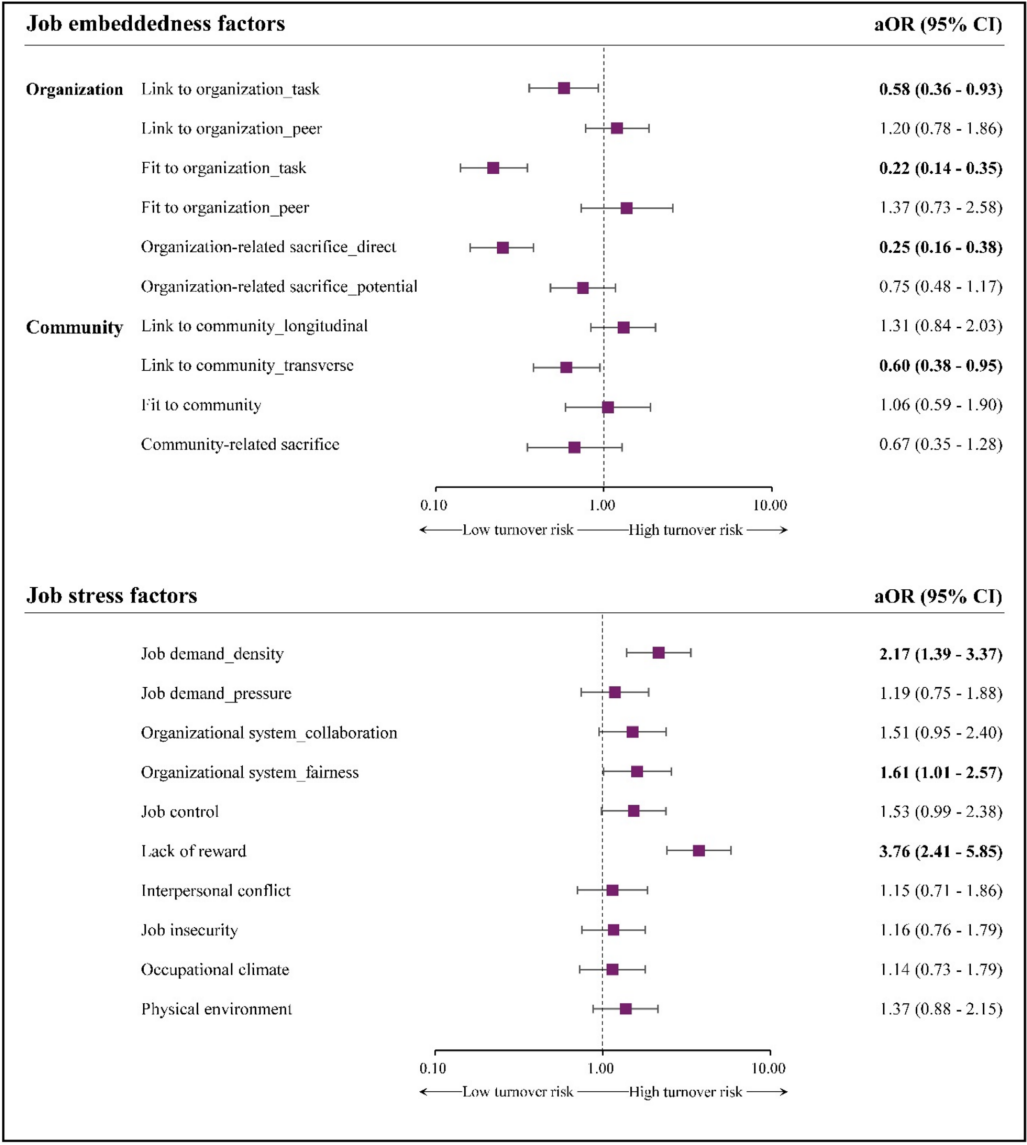
Adjusted odds ratios for job embeddedness and job stress factors associated with turnover intention. aOR, adjusted odds ratio; CI, confidence interval

Among the job stress factors, LR showed the strongest association with higher turnover intention, corresponding to an almost fourfold increase in odds, followed by JD_density and OS_fairness. Regarding job embeddedness, FO_task and OrS_direct demonstrated the strongest protective associations, with substantially lower turnover intention scores and odds, followed by LC_transverse and LO_task. These overall effect patterns were consistent across both linear and logistic regression models (Table 2 and Fig. 2).

**Table 2:** Results of multivariable linear regression analysis predicting turnover intention

Predictive factors	Coefficient (β)	SE	95% CI	*t*-value	*P* value
Job embeddedness					
Intercept	19.02	1.11			
LO_task	**− 0.14**	0.05	(**− **0.25, **− **0.04)	**− **2.65	0.01
LO_peer	0.17	0.10	(**− **0.02, 0.36)	1.80	0.07
FO_task	**− 0.34**	0.04	(**− **0.42, **− **0.27)	**− **8.94	< 0.01
FO_peer	0.19	0.11	(**− **0.03, 0.41)	1.69	0.09
OrS_direct	**− 0.29**	0.06	(**− **0.40, **− **0.18)	**− **5.09	< 0.01
OrS_potential	**− **0.04	0.05	(**− **0.14, 0.05)	**− **0.88	0.38
LC_longitudinal	0.07	0.07	(**− **0.05, 0.20)	1.14	0.26
LC_transverse	**− 0.28**	0.13	(**− **0.54, **− **0.02)	**− **2.12	0.03
FC	0.09	0.06	(**− **0.02, 0.20)	1.66	0.10
CrS	**− **0.16	0.10	(**− **0.35, 0.04)	**− **1.60	0.11
Job stress					
Intercept	**− **1.03	1.11			
JD_density	**0.16**	0.04	(0.08, 0.23)	3.96	< 0.01
JD_pressure	0.07	0.09	(**− **0.11, 0.25)	0.75	0.45
OS_collaboration	0.10	0.07	(**− **0.04, 0.24)	1.36	0.17
OS_fairness	**0.19**	0.07	(0.05, 0.33)	2.68	0.01
JC	0.04	0.06	(**− **0.07, 0.15)	0.78	0.44
LR	**0.36**	0.05	(0.26, 0.45)	7.20	< 0.01
IC	0.00	0.06	(**− **0.11, 0.12)	0.07	0.95
JI	**− **0.02	0.08	(**− **0.17, 0.13)	-0.28	0.78
OC	0.02	0.09	(**− **0.17, 0.20)	0.19	0.85
PE	0.12	0.08	(**− **0.04, 0.27)	1.44	0.15

*CI* Confidence interval, *CrS* Community-related sacrifices, *FC* Fit to community, *FO* Fit to organization, *IC* Interpersonal conflict, *JC* Job control, *JD* Job demand, *JI* Job insecurity, *LC* Link to community, *LO* Link to organization, *LR* Lack of reward, *OC* Occupational climate, *OrS* Organization-related sacrifice, *OS* Organizational system, *PE* Physical environment, *SE* Standard error

Bold values indicate statistical significance (*P* < 0.05)

Sensitivity analyses of logistic regression models using alternative thresholds to define high turnover intention (e.g., scores > 8 or > 10 instead of the median cutoff of 9) demonstrated consistent effect directions and statistical robustness for the major predictors identified in the logistic regression model using the median cutoff (FO_task, OrS_direct, LR, and JD_density). Predictors with borderline CIs in the median-cutoff logistic regression model (e.g., LO_task, OS_fairness, and OS_collaboration) showed expected variations in statistical significance across thresholds (Supplementary Table S4).

### Subgroup analysis

Subgroup analyses revealed several notable interaction patterns, particularly in terms of employment duration, hospital type, and current department (Tables 3 and 4). By employment duration, OrS_direct showed the strongest protective associations among pharmacists with < 3 or 3–10 years of employment, whereas these associations were attenuated among those with ≥ 10 years. In contrast, OrS_potential was linked to higher turnover intention among pharmacists with < 3 years of employment, but to lower turnover intention among those aged 3–10 years. According to the hospital type, FO_peer showed a distinct association with higher turnover intention in tertiary hospitals, a pattern that was not observed in general hospitals. Regarding job stress, JI was more strongly related to higher turnover intention in general dispensing departments, whereas insufficient organizational system collaboration (OS_collaboration) showed stronger associations with higher turnover intention in non-dispensing departments.

**Table 3:** Subgroup analysis of factors influencing turnover intention by hospital location, hospital type, and employment duration

Predictive factors	aOR (95% CI)						
	Metropolitan (*N* = 392)	Non-metropolitan (*N* = 200)	Tertiary hospital (*N* = 327)	General hospital (*N* = 265)	< 3 years (*N* = 204)	3–10 years (*N* = 195)	≥ 10 years (*N* = 193)
Job embeddedness							
LO_task	0.69 (0.39–1.23)	**0.28 (0.11–0.76)**	0.82 (0.43–1.57)	**0.35 (0.17–0.76)**	0.20 (0.03–1.57)	0.84 (0.36–1.97)	1.76 (0.32–9.75)
LO_peer	1.16 (0.69–1.97)	1.42 (0.59–3.42)	1.15 (0.65–2.02)	1.36 (0.65–2.85)	0.61 (0.27–1.37)	1.22 (0.56–2.62)	1.44 (0.59–3.53)
FO_task	**0.23 (0.14–0.40)**	**0.17 (0.06–0.49)**	**0.21 (0.11–0.41)**	**0.19 (0.09–0.39)**	**0.17 (0.07–0.40)**	**0.16 (0.07–0.38)**	**0.19 (0.07–0.51)**
FO_peer	1.32 (0.64–2.72)	2.02 (0.47–8.64)	2.66 (1.11–6.37)^*^	0.63 (0.23–1.78)	0.95 (0.35–2.58)	1.65 (0.53–5.10)	1.59 (0.28–9.17)
OrS_direct	**0.30 (0.18–0.50)**	**0.13 (0.05–0.33)**	**0.26 (0.14–0.49)**	**0.21 (0.11–0.42)**	0.07 (0.03–0.16)^*^	0.29 (0.13–0.63)^*^	0.51 (0.22–1.22)
OrS_potential	0.80 (0.47–1.35)	0.67 (0.27–1.63)	0.74 (0.41–1.34)	0.85 (0.41–1.73)	2.69 (1.01–7.19)^*^	0.42 (0.20–0.87)^*^	0.62 (0.25–1.52)
LC_longitudinal	1.20 (0.70–2.08)	2.02 (0.85–4.79)	1.62 (0.90–2.93)	1.03 (0.52–2.04)	1.50 (0.62–3.64)	1.16 (0.55–2.41)	1.80 (0.76–4.28)
LC_transverse	0.65 (0.37–1.15)	**0.40 (0.16–0.97)**	**0.38 (0.19–0.75)**	0.85 (0.43–1.68)	0.47 (0.13–1.67)	0.74 (0.33–1.62)	0.69 (0.32–1.53)
FC	0.80 (0.38–1.72)	1.10 (0.38–3.17)	0.98 (0.46–2.09)	1.08 (0.42–2.82)	0.71 (0.23–2.22)	1.88 (0.70–5.06)	0.43 (0.12–1.50)
CrS	1.45 (0.63–3.34)	0.11 (0.03–0.40)^*^	0.65 (0.28–1.52)	0.77 (0.27–2.20)	0.74 (0.19–2.84)	0.39 (0.14–1.05)	1.54 (0.39–6.03)
Job stress							
JD_density	**1.83 (1.09–3.09)**	**2.99 (1.21–7.37)**	**2.32 (1.27–4.24)**	**2.16 (1.10–4.24)**	1.66 (0.74–3.73)	**2.37 (1.13–4.98)**	**2.94 (1.19–7.24)**
JD_pressure	1.08 (0.62–1.88)	1.55 (0.61–3.97)	1.79 (0.96–3.35)	0.76 (0.36–1.59)	0.78 (0.28–2.18)	1.26 (0.61–2.61)	1.19 (0.48–2.96)
OS_collaboration	**1.87 (1.08–3.24)**	0.79 (0.31–2.02)	1.38 (0.74–2.57)	1.70 (0.83–3.48)	1.42 (0.57–3.56)	1.83 (0.87–3.86)	2.03 (0.80–5.19)
OS_fairness	1.43 (0.81–2.50)	1.85 (0.74–4.66)	**2.20 (1.18–4.09)**	1.12 (0.53–2.38)	**2.76 (1.18–6.43)**	1.63 (0.75–3.55)	0.72 (0.28–1.87)
JC	1.60 (0.95–2.69)	1.40 (0.55–3.59)	1.46 (0.80–2.64)	1.67 (0.82–3.39)	**2.95 (1.29–6.76)**	0.85 (0.40–1.81)	1.54 (0.63–3.77)
LR	**2.94 (1.75–4.94)**	**7.80 (3.06–19.91)**	**3.50 (1.95–6.28)**	**4.64 (2.24–9.63)**	**6.32 (2.81–14.23)**	**2.24 (1.03–4.86)**	**5.06 (2.12–12.11)**
IC	1.18 (0.66–2.11)	1.35 (0.52–3.50)	1.32 (0.69–2.54)	0.96 (0.45–2.04)	1.30 (0.51–3.29)	1.17 (0.50–2.70)	1.13 (0.46–2.75)
JI	1.04 (0.62–1.76)	1.42 (0.62–3.28)	1.01 (0.56–1.83)	1.33 (0.69–2.58)	1.07 (0.49–2.36)	1.77 (0.82–3.82)	0.67 (0.29–1.56)
OC	1.16 (0.69–1.95)	1.12 (0.42–2.98)	0.94 (0.52–1.70)	1.45 (0.71–2.97)	0.92 (0.42–2.02)	1.68 (0.81–3.49)	1.03 (0.39–2.70)
PE	1.32 (0.80–2.18)	1.43 (0.49–4.17)	1.20 (0.67–2.17)	1.53 (0.75–3.14)	0.83 (0.36–1.93)	1.37 (0.66–2.83)	2.52 (1.00–6.35)

*aOR* adjusted odds ratio, *CrS* Community-related sacrifices, *FC* Fit to community, *FO* Fit to organization, *IC* Interpersonal conflict, *JC* Job control, *JD* Job demand, *JI*Job insecurity, *LC* Link to community, *LO*Link to organization, *LR* Lack of reward, *OC* Occupational climate, *OrS* Organization-related sacrifice, *OS* Organizational system, *PE *Physical environment

Bold indicates statistically significant subgroup interaction (*P* < 0.05)

**Table 4:** Subgroup analysis of factors influencing turnover intention by current department, sex, and marital status

Predictive factors	aOR (95% CI)					
	General dispensing (*N* = 273)	Non-general dispensing (*N* = 319)	Women (*N* = 518)	Men (*N* = 74)	Unmarried (*N* = 299)	Married (*N* = 293)
Job embeddedness						
LO_task	0.54 (0.25–1.17)	0.62 (0.33–1.16)	**0.53 (0.32–0.88)**	0.98 (0.14–7.05)	0.54 (0.26–1.11)	**0.49 (0.24–0.98)**
LO_peer	1.48 (0.79–2.77)	1.10 (0.58–2.09)	1.24 (0.78–1.97)	0.38 (0.06–2.31)	1.11 (0.61–2.04)	1.15 (0.58–2.28)
FO_task	**0.22 (0.11–0.44)**	**0.19 (0.10–0.38)**	**0.23 (0.14–0.38)**	0.16 (0.02–1.34)	**0.18 (0.09–0.35)**	**0.26 (0.13–0.52)**
FO_peer	1.39 (0.55–3.51)	1.89 (0.75–4.79)	1.19 (0.59–2.38)	2.79 (0.25–30.70)	1.55 (0.68–3.52)	1.32 (0.45–3.87)
OrS_direct	**0.16 (0.08–0.32)**	**0.35 (0.19–0.64)**	**0.24 (0.15–0.39)**	**0.11 (0.01–0.89)**	**0.17 (0.09–0.32)**	**0.36 (0.18–0.71)**
OrS_potential	0.94 (0.49–1.81)	0.58 (0.31–1.09)	0.84 (0.53–1.34)	0.45 (0.07–2.75)	0.98 (0.52–1.85)	0.56 (0.29–1.08)
LC_longitudinal	1.49 (0.78–2.82)	1.11 (0.59–2.12)	1.37 (0.86–2.17)	0.71 (0.12–4.21)	1.55 (0.83–2.89)	1.31 (0.67–2.55)
LC_transverse	0.68 (0.32–1.43)	0.55 (0.29–1.02)	0.61 (0.37–1.00)	0.33 (0.05–2.13)	NA	0.57 (0.31–1.05)
FC	1.56 (0.64–3.81)	0.76 (0.33–1.71)	0.99 (0.53–1.84)	4.12 (0.47–36.29)	1.21 (0.54–2.71)	0.86 (0.35–2.11)
CrS	0.29 (0.11–0.76)^*^	1.54 (0.60–3.94)	0.84 (0.42–1.67)	0.03 (0.002–0.65)^*^	0.64 (0.25–1.60)	0.69 (0.27–1.81)
Job stress						
JD_density	**2.84 (1.46–5.53)**	1.78 (0.93–3.41)	**1.96 (1.24–3.11)**	6.24 (0.96–40.83)	1.37 (0.73–2.58)	**3.79 (1.91–7.51)**
JD_pressure	1.16 (0.55–2.47)	1.35 (0.71–2.55)	1.26 (0.78–2.04)	0.78 (0.10–6.06)	1.38 (0.70–2.75)	1.02 (0.51–2.04)
OS_collaboration	0.85 (0.42–1.73)	3.02 (1.54–5.92)^*^	1.55 (0.96–2.51)	0.9 (0.12–6.65)	1.32 (0.66–2.62)	2.21 (1.13–4.32)^*^
OS_fairness	1.89 (0.94–3.78)	1.09 (0.54–2.18)	1.58 (0.97–2.59)	1.69 (0.24–11.88)	**2.36 (1.25–4.45)**	0.90 (0.43–1.89)
JC	1.85 (0.94–3.63)	1.43 (0.76–2.69)	1.44 (0.90–2.29)	4.19 (0.73–24.00)	1.54 (0.84–2.83)	1.50 (0.74–3.01)
LR	**5.51 (2.79–10.87)**	**3.68 (1.92–7.04)**	**3.55 (2.24–5.63)**	**8.64 (1.12–66.68)**	**4.25 (2.30–7.86)**	**3.20 (1.61–6.33)**
IC	1.84 (0.88–3.82)	0.68 (0.34–1.38)	1.18 (0.71–1.95)	0.79 (0.09–6.63)	1.15 (0.57–2.31)	1.11 (0.55–2.24)
JI	2.21 (1.13–4.30)^*^	0.63 (0.33–1.19)	1.15 (0.73–1.83)	1.24 (0.23–6.54)	0.75 (0.40–1.40)	2.00 (1.05–3.78)^*^
OC	0.86 (0.43–1.72)	1.62 (0.85–3.09)	1.08 (0.67–1.73)	2.38 (0.40–14.22)	1.07 (0.58–2.00)	1.46 (0.72–2.96)
PE	1.53 (0.78–2.98)	1.35 (0.69–2.63)	1.43 (0.90–2.27)	0.94 (0.10–8.62)	1.31 (0.72–2.37)	1.91 (0.91–3.98)

*aOR* adjusted odds ratio, *CrS* Community-related sacrifices, *FC* Fit to community, *FO* Fit to organization, *IC* Interpersonal conflict, *JC* Job control, *JD* Job demand, *JI* Job insecurity, *LC* Link to community, *LO* Link to organization, *LR* Lack of reward, *OC* Occupational climate, *OrS* Organization-related sacrifice, *OS* Organizational system, *PE* Physical environment

Bold indicates statistically significant subgroup interaction (*P* < 0.05)

## Discussion

### Turnover intention and key predictors in the overall sample

In this nationwide survey of Korean hospital pharmacists, 43.1% were classified as having high turnover intention. This figure lies at the upper end of international reports (25.0–68.7%) [8] and indicates a substantial workforce stability concern within the Korean hospital pharmacy sector. In this study, job embeddedness components (FO_task, OrS_direct, LO_task, LC_transverse) reduced turnover intention, while job stress factors (LR, JD_density, OS_fairness) increased it. These associations were consistently observed not only in the multivariable logistic regression analysis using the binary outcome variable, but also in the multivariable linear regression analysis using continuous variables. Additionally, subgroup analyses clarified the interaction of individual and organizational factors.

International studies have reported similar predictors of turnover intention among pharmacists and other health professionals, including low job satisfaction, low organizational commitment, role-related stressors, inadequate compensation, and heavy workload [8, 42, 43]. These international findings parallel our results, in which a lack of rewards, high job demands, and perceived organizational unfairness were the key determinants of turnover intention. The results of job suitability and role commitment (FO_task, LO_task) align with prior evidence that individuals who fit well with their organization are more likely to remain [44], suggesting that job–person fit is key to workforce planning. In contrast, JD_density increases turnover intention. OrS_direct and LR, which reflect role-related rewards and respect, significantly influenced turnover intention, with LR being the strongest predictor. Similar patterns have been reported internationally, with studies showing that U.S. pharmacists reported higher job satisfaction when they worked fewer hours (aOR 2.91) and earned a higher income (aOR 4.60) [43]. In addition, a meta-analysis found that the “sacrifice” dimension showed a strong negative correlation with turnover intention (*r* = –0.460), supporting our finding that direct organizational sacrifices (OrS_direct) are closely tied to retention [45]. Taken together, these similarities suggest that the patterns observed in Korean hospital pharmacists may reflect broader retention challenges reported in other healthcare settings.

### Differential associations by career stage, department, and hospital type

By employment duration, early-career pharmacists (< 3 years) showed stronger associations with organizational fairness, direct organizational sacrifice (OrS_direct), rewards (LR), and job autonomy (JC). OrS_direct was a major determinant in this group, with LR demonstrating the largest effect. These results may be interpreted in light of the younger generations’ tendency toward present-oriented value over future benefits [46]*.* The association with job autonomy may be related to the challenges junior staff encounter in hierarchical and standardized work environments that restrict decision-making authority [47, 48]*.* Together with the heightened impact of OS_fairness observed in the < 3-year group, this suggests that the fair provision of direct rewards may play a crucial role in curbing turnover intention among early-career employees.

OrS_potential was associated with lower turnover intention among those with 3–10 years of practice. In addition, job demand-related pressures were more prominent among mid-career pharmacists (3–10 years). Although the interaction was not statistically significant, subgroup analysis indicated that JD_density was associated with higher turnover intention in women, married, and experienced pharmacists (≥ 3 years of experience), reaffirming that workload with domestic responsibilities (e.g., childcare) impacts turnover intention [49, 50]*.* Taken together, these findings suggest that mitigating workload intensity and supporting work–family balance may be particularly important for reducing turnover intentions among mid-career hospital pharmacists.

In non-dispensing departments, OS_collaboration was associated with higher turnover intention. This pattern suggests that collaborative expectations in clinical and inpatient environments can generate additional interpersonal or coordination burdens, potentially increasing rather than alleviating stress. Alternatively, pharmacists in these roles may have heightened exposure to interprofessional dynamics or organizational pressures that render collaboration more demanding, thereby amplifying dissatisfaction and turnover intention. These role-specific patterns suggest that tailored organizational management strategies, including job rotation or role-adjusted support, may be needed to mitigate turnover intention across different departments.

With respect to hospital type, certain predictors demonstrated distinct patterns in tertiary hospitals compared to general hospitals. Notably, FO_peer showed an inverse association only in tertiary institutions. This suggests that in large, highly specialized settings where workflow complexity and interdisciplinary interactions are more intensive, coworker-related embeddedness may operate differently than in general hospitals. [51]. Other factors related to organizational fit, direct rewards, and workload (e.g., OrS_direct and JD_density) may similarly be subject to contextual influences specific to tertiary care environments.

### Interpretation of the factor structure

The observed factor structure may also be influenced by well-established methodological characteristics of factor analysis, such as the small number of items within certain subdimensions. Even with these methodological considerations, some of the observed patterns may be tentatively interpreted in relation to the generational characteristics discussed in previous literature. Several factor splits, such as task- versus colleague-oriented components (LO/FO), collaboration versus fairness (OS), and direct versus potential sacrifices (OrS), may be consistent with patterns reported among younger generations, including a greater emphasis on autonomy, fairness, and immediate rewards over future benefits [52, 53]; however, these interpretations remain hypothetical and warrant further empirical investigation.

Alternative explanations for this finding should be considered. Structural characteristics of Korean pharmacy practice—particularly the high dispensing workload, limited staffing, and the still underrecognized clinical responsibilities of hospital pharmacists—may shape how job-related items are interpreted [54]. As clinical pharmacy roles are largely restricted to hospitals in Korea, this structural divide likely contributes to the distinct separation of task-related components. The grouping of compensation-related items may also reflect a lack of direct financial reimbursement for clinical pharmacy services, where the imbalance between the clinical workload and limited remuneration further reinforces the separation of sacrifice-related components.

### Strengths and limitations

This study has several strengths. It comprehensively evaluated the factors associated with hospital pharmacists’ turnover intentions by integrating job embeddedness and job stress models, emphasizing organizational and community embeddedness as well as work-related stress—factors often overlooked in prior research. Also, a nationally representative sample (*n* = 592) aligned with the national hospital pharmacist workforce. Subgroup analyses revealed distinct patterns across demographic and institutional characteristics, with early career pharmacists showing particularly high turnover intention due to inadequate compensation, limited autonomy, and organizational unfairness. These findings reinforce previous research and highlight the need for organizational strategies to improve pharmacist retention by considering generational characteristics and differences [55, 56]*.*

Despite these strengths, some limitations exist. As a survey-based study, it is susceptible to response and non-response bias. Although the overall response rate was modest (16.0%), the demographic and institutional distributions of respondents were broadly similar to those of national KSHP workforce statistics, suggesting reasonable representativeness. Nonetheless, the modest response rate and lack of information on non-respondents introduce the possibility of non-response bias and may limit the generalizability of the findings. Factor-level heterogeneity warrants careful interpretation. Two job embeddedness factors demonstrated Cronbach’s *α* < 0.6, likely due to the restricted number of items; however, such low reliability may weaken the precision of estimated associations. For the LC factor, culturally less relevant items from Mitchell’s questionnaire (e.g., “Does your spouse work outside the home?”) were excluded. The CrS included all items, but exhibited heterogeneity owing to fewer contextually relevant questions (e.g., “My neighborhood is safe”), which may have further contributed to reduced internal consistency. In the LC domain, items employed mixed response formats: binary and three-level. Although this heterogeneity could affect item weighting, mixed response formats were retained to minimize extreme value distortions and preserve the theoretical meaning of social proximity, a core component of community embeddedness. Nevertheless, these mixed formats may still reduce comparability across items and introduce additional uncertainty into factor estimation [22, 57]*.* Although dichotomizing predictors at the median has been commonly employed in prior studies, this approach inevitably compresses the variability inherent in continuous measures and may limit sensitivity [20, 39, 41]*.* Accordingly, linear regression models with continuous predictors served as the primary analytical framework, and logistic regression models based on median splits were interpreted as supplementary policy-oriented summaries. We also examined alternative cut-offs for defining high turnover intention, which yielded similar directions and magnitudes of association for the main predictors. To address concerns about arbitrariness of a median split, we confirmed consistency of associations using multivariable linear regression. Finally, although turnover intention is strongly associated with actual turnover [21]*,* as only turnover intention was measured, longitudinal studies are needed to validate predictive thresholds with actual turnover.

## Conclusions

This study identified key predictors of turnover intention among hospital pharmacists in Korea. Strong job embeddedness—particularly organizational fit and links—was associated with lower turnover intention, whereas lack of rewards, high workloads, and perceived organizational unfairness significantly increased it. The predictors identified in this study point to the need for several practical strategies that may help improve retention, especially for early career hospital pharmacists. Equitable compensation may help address dissatisfaction with rewards, whereas balanced workloads can help reduce pressure from high job demands. Transparent advancement processes may ease perceptions of unfairness within organizations. In addition, better alignment between roles and competencies may strengthen job embeddedness and support overall workforce stability. Future research should employ longitudinal designs to clarify the causal pathways between turnover intention and actual turnover and evaluate targeted interventions for the modifiable workplace determinants identified in this study. Intervention studies testing specific retention strategies, such as mentorship programs, flexible scheduling, and compensation reform, would further strengthen the practical relevance of these findings.

## Supplementary Information

Supplementary Material 1.

## Data Availability

Datasets used and/or analyzed in this study can be obtained from the corresponding author upon request.

## Abbreviations

aOR: Adjusted odds ratio
CI: Confidence interval
CrS: Community-related sacrifice
CVI: Content validity index
EFA: Exploratory factor analysis
FC: Fit to community
FO: Fit to organization
IC: Interpersonal conflict
IRB: Institutional Review Board
JC: Job control
JD: Job demand
JI: Job insecurity
KOSS: Korean Occupational Stress Scale
KSHP: Korean Society of Health-System Pharmacists
LC: Link to community
LO: Link to organization
LR: Lack of reward
NHS: National Health Service
OC: Occupational climate
OrS: Organization-related sacrifice
OS: Organizational system
PE: Physical environment
STROBE: Strengthening the Reporting of Observational Studies in Epidemiology
VIF: Variance inflation factor
