# Supplementary material for: A nationwide cross-sectional survey on factors affecting turnover intention among hospital pharmacists
Source: Hum Resour Health. 2026 Feb 7;24:10. doi: 10.1186/s12960-026-01052-4 (PMC12895604; doi:10.1186/s12960-026-01052-4)
Supplement: Supplementary file 1 — Supplementary Material 1. [file 12960_2026_1052_MOESM1_ESM.docx]

**Supplementary Materials**

**Table S1.** Demographic and institutional characteristics of survey respondents compared with the national hospital pharmacist workforce

**Table S2.** Factor loading on the items for turnover intention, job embeddedness and job stress

**Table S3.** Internal consistency on the items for turnover intention, job embeddedness and job stress

**Table S4.** Sensitivity analysis of adjusted odds ratios for job embeddedness and job stress factors by different turnover intention score thresholds (N = 592)

**Table S1. Demographic and institutional characteristics of survey respondents compared with the national hospital pharmacist workforce**

| **Characteristics** | | **Survey respondents**  **(n = 592)** | **National hospital pharmacist workforce (n = 3,708)** |
| --- | --- | --- | --- |
| Sex | Female | 518 (87.5) | 3315 (89.4) |
|  | Male | 74 (12.5) | 393 (10.6) |
| Age group, years | < 30 | 102 (17.2) | 544 (14.7) |
|  | 30–40 | 288 (48.6) | 1849 (49.9) |
|  | 40–50 | 129 (21.8) | 715 (19.3) |
|  | ≥ 50 | 73 (12.3) | 600 (16.2) |
| Hospital location | Capital region | 246 (41.6) | 1513 (40.8) |
|  | Metropolitan cities | 146 (24.7) | 849 (22.9) |
|  | Provinces | 200 (33.7) | 1346 (36.3) |
| Hospital type | Tertiary hospital | 327 (55.2) | 2153 (58.0) |
|  | General hospital | 265 (44.8) | 1555 (42.0) |

**Table S2. Factor loading on the items for turnover intention, job embeddedness and job stress**

| **Items** | | **Total correlation coefficient** | **Factor 1** | **Factor 2** |
| --- | --- | --- | --- | --- |
| **Turnover intention** | | | | |
|  | Thoughts of turnover | 0.61 | **0.80** |  |
|  | Turnover plan within a year | 0.85 | **0.94** |  |
|  | Turnover expectation within a year | 0.85 | **0.94** |  |
| Eigenvalue | |  | 2.42 |  |
| Cumulative variance explained (%) | |  | 80.65 |  |
| **Job embeddedness** | | | | |
| Link to organization (LO) | |  |  |  |
| *task* | Number of assigned tasks | 0.26 | **0.50** | −0.02 |
|  | Number of work committees | 0.44 | **0.67** | 0.20 |
|  | Years of hospital practice | 0.53 | **0.89** | −0.16 |
|  | Employment duration at the current hospital | 0.55 | **0.88** | −0.09 |
| *peer* | Number of regularly interacting colleagues | −0.02 | −0.14 | **0.81** |
|  | Number of dependable colleagues | 0.15 | 0.10 | **0.85** |
| Eigenvalue | |  | 2.31 | 1.43 |
| Cumulative variance explained (%) | |  | 38.58 | 62.34 |
| Fit to organization (FO) | |  |  |  |
| *task* | Job fit | 0.51 | **0.59** | 0.20 |
|  | Department adaptability | 0.74 | **0.73** | 0.38 |
|  | Organizational culture fit | 0.70 | **0.65** | 0.47 |
|  | Authority and responsibility satisfaction | 0.67 | **0.75** | 0.21 |
|  | Value alignment | 0.65 | **0.75** | 0.18 |
|  | Goal achievement feasibility | 0.67 | **0.84** | 0.02 |
|  | Professional growth satisfaction | 0.65 | **0.82** | 0.03 |
| *peer* | Positive feelings toward colleagues | 0.42 | 0.16 | **0.82** |
|  | Similarity with colleagues | 0.36 | 0.11 | **0.81** |
| Eigenvalue | |  | 4.42 | 1.21 |
| Cumulative variance explained (%) | |  | 49.11 | 62.55 |
| Organization-related sacrifice (OrS) | |  |  |  |
| *direct* | Work autonomy | 0.39 | **0.64** | 0.09 |
|  | Interdepartmental respect | 0.45 | **0.71** | 0.11 |
|  | Perceived loss upon leaving the hospital | 0.34 | **0.49** | 0.17 |
|  | Promotion opportunities | 0.43 | **0.65** | 0.16 |
|  | Compensation appropriateness | 0.59 | **0.67** | 0.36 |
| *potential* | Welfare benefits satisfaction | 0.62 | 0.34 | **0.73** |
|  | Medical benefits satisfaction | 0.47 | 0.12 | **0.76** |
|  | Retirement benefits satisfaction | 0.53 | 0.23 | **0.74** |
|  | Job security prospects | 0.35 | 0.09 | **0.58** |
| Eigenvalue | |  | 3.32 | 1.08 |
| Cumulative variance explained (%) | |  | 36.96 | 48.94 |
| Link to community (LC) | |  |  |  |
| *longitudinal* | Childhood | 0.40 | **0.82** | 0.02 |
|  | Family | 0.46 | **0.79** | 0.27 |
|  | Friends | 0.17 | **0.62** | −0.20 |
| *transverse* | Home ownership | 0.23 | 0.05 | **0.84** |
|  | Marital status | 0.18 | -0.03 | **0.86** |
| Eigenvalue | |  | 1.76 | 1.49 |
| Cumulative variance explained (%) | |  | 35.15 | 64.93 |
| Fit to community (FC) | |  |  |  |
|  | Residential satisfaction | 0.72 | **0.84** |  |
|  | Weather suitability | 0.57 | **0.72** |  |
|  | Community compatibility | 0.77 | **0.87** |  |
|  | Community familiarity | 0.68 | **0.81** |  |
|  | Availability of leisure activities | 0.59 | **0.73** |  |
| Eigenvalue | |  | 3.17 |  |
| Cumulative variance explained (%) | |  | 63.35 |  |
| Community-related sacrifice (CrS) | |  |  |  |
|  | Leaving difficulty | 0.38 | **0.73** |  |
|  | Respect | 0.39 | **0.75** |  |
|  | Safety | 0.36 | **0.72** |  |
| Eigenvalue | |  | 1.61 |  |
| Cumulative variance explained (%) | |  | 53.67 |  |
| **Job stress** | | | | |
| Job demand (JD) | |  |  |  |
| *density* | Time pressure | 0.66 | **0.81** | 0.20 |
|  | Interruptions | 0.63 | **0.64** | 0.40 |
|  | Focus | 0.48 | **0.55** | 0.27 |
|  | Rest | 0.50 | **0.79** | −0.06 |
|  | Multitasking | 0.58 | **0.55** | 0.46 |
|  | Work-life balance | 0.58 | **0.73** | 0.18 |
| *pressure* | Responsibility | 0.48 | 0.28 | **0.69** |
|  | Job burden | 0.35 | 0.01 | **0.86** |
| Eigenvalue | |  | 3.55 | 1.07 |
| Cumulative variance explained (%) | |  | 44.42 | 57.74 |
| Organizational system (OS) | |  |  |  |
| *collaboration* | Work support adequacy | 0.48 | **0.71** | 0.19 |
|  | Interdepartmental cooperation | 0.42 | **0.76** | 0.05 |
|  | Cooperation within hospital | 0.59 | **0.76** | 0.28 |
| *fairness* | Communication opportunity | 0.45 | 0.40 | **0.46** |
|  | Career and promotion prospects | 0.51 | 0.17 | **0.77** |
|  | Position property | 0.44 | 0.04 | **0.82** |
|  | Personnel system fairness | 0.57 | 0.40 | **0.63** |
| Eigenvalue | |  | 2.99 | 1.02 |
| Cumulative variance explained (%) | |  | 42.65 | 57.20 |
| Job control (JC) | |  |  |  |
|  | Decision making authority | 0.50 | **0.75** |  |
|  | Workload and schedule control | 0.47 | **0.73** |  |
|  | Creativity requirement | 0.50 | **0.74** |  |
|  | High-level skill and knowledge necessity | 0.37 | **0.61** |  |
| Eigenvalue | |  | 2.04 |  |
| Cumulative variance explained (%) | |  | 50.89 |  |
| Lack of reward (LR) | |  |  |  |
|  | Job satisfaction | 0.57 | **0.75** |  |
|  | Compensation adequacy | 0.32 | **0.47** |  |
|  | Respect and trust | 0.45 | **0.63** |  |
|  | Work interest | 0.55 | **0.75** |  |
|  | Workplace improvement expectations | 0.47 | **0.65** |  |
|  | Skill development and application opportunities | 0.54 | **0.73** |  |
| Eigenvalue | |  | 2.69 |  |
| Cumulative variance explained (%) | |  | 44.82 |  |
| Interpersonal conflict (IC) | |  |  |  |
|  | Supervisor support | 0.33 | **0.53** |  |
|  | Peer support | 0.54 | **0.74** |  |
|  | Colleague understanding | 0.63 | **0.85** |  |
|  | Colleague sharing | 0.59 | **0.83** |  |
| Eigenvalue | |  | 2.25 |  |
| Cumulative variance explained (%) | |  | 56.27 |  |
| Job insecurity (JI) | |  |  |  |
|  | Future uncertainty | 0.58 | **0.89** |  |
|  | Work condition concerns | 0.58 | **0.89** |  |
| Eigenvalue | |  | 1.58 |  |
| Cumulative variance explained (%) | |  | 78.83 |  |
| Occupational climate (OC) | |  |  |  |
|  | Task inconsistency | 0.45 | **0.85** |  |
|  | Department overcontrol | 0.45 | **0.85** |  |
| Eigenvalue | |  | 1.45 |  |
| Cumulative variance explained (%) | |  | 72.48 |  |
| Physical environment (PE) | |  |  |  |
|  | Work method risks | 0.48 | **0.86** |  |
|  | Physical strain | 0.48 | **0.86** |  |
| Eigenvalue | |  | 1.48 |  |
| Cumulative variance explained (%) | |  | 73.91 |  |
| Item loading > 0.4 is indicated in bold. | | | | |

**Table S3. Internal consistency on the items for turnover intention, job embeddedness and job stress**

| **Classification** | | **Number of items** | **Cronbach’s α coefficients ^a^** |
| --- | --- | --- | --- |
|  | Turnover intention | 3 | 0.88 |
| **Job embeddedness** | | | |
|  | Link to organization (LO)_task | 4 | 0.74 |
|  | Fit to organization (FO)_task | 7 | 0.88 |
|  | Organization-related sacrifice (OrS)_direct | 5 | 0.67 |
|  | Organization-related sacrifice (OrS)_potential | 4 | 0.71 |
|  | Link to community (LC)_longitudinal | 3 | 0.56 |
|  | Fit to community (FC) | 5 | 0.85 |
|  | Community-related sacrifices (CrS) | 3 | 0.55 |
| **Job stress** | | | |
|  | Job demand (JD)_density | 6 | 0.82 |
|  | Organizational system (OS)_collaboration | 3 | 0.67 |
|  | Organizational system (OS)_fairness | 4 | 0.70 |
|  | Job control (JC) | 4 | 0.68 |
|  | Lack of reward (LR) | 6 | 0.74 |
|  | Interpersonal conflict (IC) | 4 | 0.72 |

^a^ Cronbach's α coefficients were examined for categories with at least three items.

**Table S4. Sensitivity analysis of adjusted odds ratios for job embeddedness and job stress factors by different turnover intention score thresholds (N = 592)**

| **Predictive factors** | | **aOR (95% CI)** | | |
| --- | --- | --- | --- | --- |
|  |  | **TI score > 8**  **(lower threshold)** | **TI score > 9 (median cutoff; main analysis)** | **TI score > 10**  **(higher threshold)** |
| **Job embeddedness** | | | | |
|  | LO_task | 0.64 (0.39–1.03) | **0.58 (0.36–0.93)** | **0.58 (0.35–0.96)** |
|  | LO_peer | 1.23 (0.79–1.90) | 1.20 (0.78–1.86) | 1.17 (0.74–1.85) |
|  | FO_task | **0.23 (0.15–0.36)** | **0.22 (0.14–0.35)** | **0.23 (0.13–0.41)** |
|  | FO_peer | 1.20 (0.64–2.27) | 1.37 (0.73–2.58) | 1.77 (0.92–3.39) |
|  | OrS_direct | **0.27 (0.18–0.42)** | **0.25 (0.16–0.38)** | **0.24 (0.14–0.40)** |
|  | OrS_potential | 0.72 (0.47–1.10) | 0.75 (0.48–1.17) | 0.64 (0.39–1.03) |
|  | LC_longitudinal | **1.64 (1.05–2.57)** | 1.31 (0.84–2.03) | 0.77 (0.48–1.23) |
|  | LC_transverse | **0.63 (0.40–1.00)** | **0.60 (0.38–0.95)** | **0.41 (0.25–0.69)** |
|  | FC | 1.18 (0.66–2.11) | 1.06 (0.59–1.90) | 1.09 (0.58–2.04) |
|  | CrS | 0.70 (0.37–1.35) | 0.67 (0.35–1.28) | 1.13 (0.57–2.24) |
| **Job stress** | | | | |
|  | JD_density | **2.38 (1.52–3.73)** | **2.17 (1.39–3.37)** | **1.65 (1.02–2.66)** |
|  | JD_pressure | 0.96 (0.60–1.53) | 1.19 (0.75–1.88) | 1.04 (0.64–1.70) |
|  | OS_collaboration | 1.55 (0.97–2.47) | 1.51 (0.95–2.40) | **1.82 (1.11–3.00)** |
|  | OS_fairness | 1.55 (0.97–2.48) | **1.61 (1.01–2.57)** | **1.91 (1.14–3.20)** |
|  | JC | 1.43 (0.91–2.23) | 1.53 (0.99–2.38) | 1.21 (0.75–1.95) |
|  | LR | **3.61 (2.29–5.71)** | **3.76 (2.41–5.85)** | **3.91 (2.40–6.39)** |
|  | IC | 1.12 (0.68–1.85) | 1.15 (0.71–1.86) | 1.30 (0.79–2.14) |
|  | JI | 1.36 (0.88–2.10) | 1.16 (0.76–1.79) | 0.85 (0.54–1.35) |
|  | OC | 1.34 (0.85–2.11) | 1.14 (0.73–1.79) | 0.87 (0.53–1.41) |
|  | PE | 1.22 (0.76–1.95) | 1.37 (0.88–2.15) | 1.47 (0.93–2.33) |

aOR, adjusted odds ratio; CI, Confidence interval; CrS, Community-related sacrifices; FC, Fit to community; FO, Fit to organization; IC, Interpersonal conflict; JC, Job control; JD, Job demand; JI, Job insecurity; LC, Link to community; LO, Link to organization; LR, Lack of reward; OC, Occupational climate; OrS, Organization-related sacrifice; OS, Organizational system; PE, Physical environment; TI, Turnover intention;
